# The complete chloroplast genome of an economic and ecological plant, paper mulberry (*Broussonetia kazinoki* × *Broussonetia papyifera*)

**DOI:** 10.1080/23802359.2017.1419088

**Published:** 2017-12-20

**Authors:** Zhenggang Xu, Guiyan Yang, Meng Dong, Liang Wu, Wan Zhang, Yunlin Zhao

**Affiliations:** a Hunan Research Center of Engineering Technology for Utilization of Environmental and Resources Plant, Central South University of Forestry and Technology, Changsha, China;; b School of Chemical and Material Engineering, Hunan City University, Yiyang, China;; c College of Forestry, Northwest A&F University, Yangling, China

**Keywords:** Paper mulberry, chloroplast genome, Illumina sequencing, *Moraceae*

## Abstract

The paper mulberry, *Broussonetia kazinoki* × *Broussonetia papyifera*, is a species within the family *Moraceae*, also is an economic and ecological plant. Complete chloroplast genome of paper mulberry was reported in this study. The total genome size was 160,903 bp in length, which is separated by a large single copy (LSC) region and a small single copy (SSC) region (89,220 and 20,079 bp in length, respectively). There were a total of 134 predicted functional genes, including 80 protein-coding genes (PCGs), eight ribosomal RNA (rRNA) genes, and 46 transfer RNA (tRNA) genes. The overall G + C content of the paper mulberry chloroplast genome was 35.74%, while the corresponding values of the LSC, SSC, and IR regions are 33.33%, 28.50%, and 42.72%, respectively. The phylogenetic analysis between *Moraceae* trees showed chloroplast genome of paper mulberry was closely related to *Morus multicaulis*.

The paper mulberry, hybridized by *Broussonetia kazinoki* × *Broussonetia papyifera*, is a species of *Moraceae*, which is distributed in Eastern Asia and pacific countries (Xianjun et al. 2014). The species has important economic values and ecological values. The bark is often used to make paper and cloth (Saito et al. 2009). The leaves have been used as a kind of forage for livestock due to the high contents of protein. The abundant flavonoids in paper mulberry have long been used in Chinese medicine (Sun et al. 2012). In terms of ecological values, the tree grow fast and have strong tolerance to cold, drought and salt (Li et al. 2011; Sun et al. 2014; Peng et al. 2015), which can be planted as a pioneer tree in ecological degradation area. Thus, it is useful to know more genetic information about paper mulberry to reveal the mechanism of evolution or adoption.

The whole genomic DNA was extracted from fresh leaves of paper mulberry seedlings with the DNeasy Plant Mini Kit (Qiagen, Valencia, CA). The seedlings were collected from Yuexi, China (115°55′–116°33′E, 30°39′–31°11′N). The specimen was kept in the laboratory at −80 °C under the accession number 20170520GS. DNA was shared by Covaris M220 (Covaris, Woburn, MA), yielding fragments of 500 bp in length. Then, an Illumina pair-endlibrary was constructed and sequenced by the Illumina HiSeq 4000 platform. After filtered out the low-quality reads by Trimmomatic V0.32 (Bolger et al. 2014), the clean reads were used for the chloroplast genome assembly by velvet v1.2.07 (Zerbino and Birney 2008). Sequences were annotated in DOGMA (Wyman et al. 2004). A circular map of the plastome was generated using OGDRAW (Lohse et al. 2007). The complete chloroplast sequence of paper mulberry has been submitted to GenBank with the accession number of MF496038.

The complete chloroplast genome of paper mulberry is 160,903 bp in length, comprising a large single copy (LSC) region of 89,220 bp and a small single copy (SSC) region of 20,079 bp, separated by two inverted repeat regions (IRs) of 25,802 bp. The overall G + C content of chloroplast genome is 35.74%, while the corresponding values of the LSC, SSC, and IRs regions are 33.33%, 28.50%, and 42.72%, respectively. The chloroplast genome contains a total of 134 predicted functional genes, including 80 protein-coding genes (PCGs), eight ribosomal RNA (rRNA) genes, and 46 transfer RNA (tRNA) genes. Among the annotated genes, nine of them (*atpF*, *rpoC1*, *rpl2* × 2, *ndhB* × 2, *ndhA*, *ycf1*, and *trnL-TAA*) contain one intron, and two genes (*ycf3* and *clpP*) include two introns.

To ascertain phylogenetic position of paper mulberry, 21 complete chloroplast genomes sequences of Moraceae family were employed and phylogenetic analysis showed that paper mulberry was clustered into *Morus* and is closely related to *Morus mongolica* (Figure 1). The complete chloroplast genome information reported here provides a useful tool not only for high quality gene mining of paper mulberry, but also for phylogenetic and evolutionary studies within *Moraceae*.

**Figure 1.**
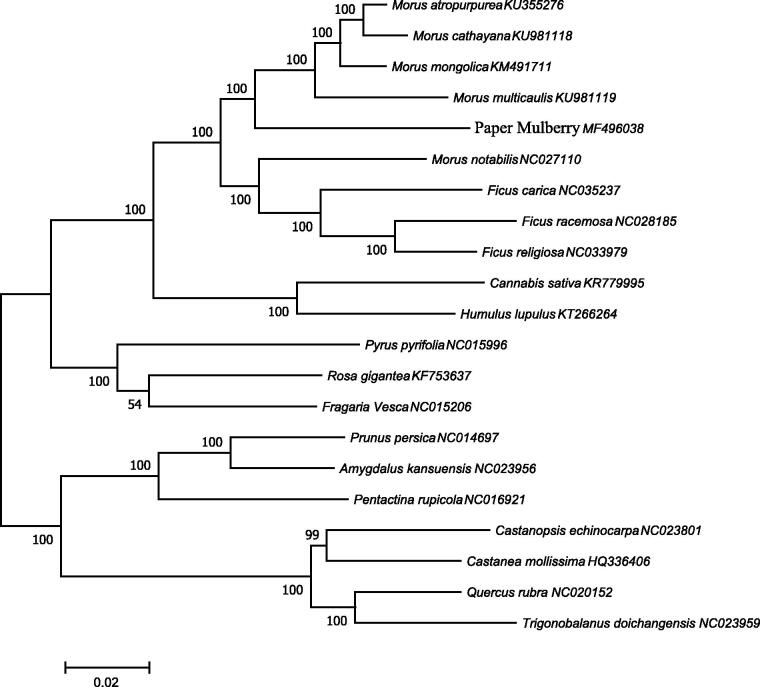
The phylogenetic tree based on 21 complete chloroplast genome sequences of *Mor*aceae family. The neighbour-joining (NJ) phylogenetic tree was constructed with MEGA 7 (with 1000 bootstrap replicates) using 80 protein-coding genes of 21 species of Moraceae family.
